# *The Organon* and Materia Medica Club of the Bay Cities of California

**Published:** 1896-09

**Authors:** W. E. Ledyard

**Affiliations:** Secretary


### *THE ORGANON* AND MATERIA MEDICA CLUB OF THE BAY CITIES OF CALIFORNIA.

The regular semi-monthly meeting was held at the office of Dr. J. M. Selfridge, in Oakland, Friday evening, April 17th, 1896.

Members present: Drs. J. M. Selfridge, W. E. Ledyard, M. T. Wilson, G. J. Augur, G. W. Swayze, C. M. Selfridge, and George H. Martin.

The meeting was called to order at 8.15 o'clock by the President, Dr. J. M. Selfridge. The minutes of the previous meeting were read by the Secretary, and, after a correction by Dr. Selfridge, were approved.

Dr. Ledyard read a letter from Dr. T. Franklin Smith, Chairman of the Committee of Organization, Registration, and Statistics, of the American Institute of Homœopathy, requesting him to have an accompanying blank filled out. Dr. Wilson moved that the letter be received and placed on file, and that the Secretary fill out the blank and send it to Dr. Smith. So ordered by the President.

The President then stated that the new publication of the *Chronic Diseases* had arrived, and he wanted to know if the members wished to commence the reading of it, or finish the reading of *The Organon* before taking up the *Chronic Diseases*. Dr. Wilson moved to continue *The Organon*. Seconded by Dr. C. M. Selfridge. Carried.

Dr. Augur then stated that he wished to hear the opinion of some of the members regarding a little matter that often puzzled him. He said: I am often asked by my patients what remedy I have given them. I often refuse to tell them, and they seem very much surprised, because they say other physicians would tell them. I do not think it right to tell patients what remedy you give them.

Dr. Martin—I tell them that I will let them know after a while. I then put it off as long as possible, and when I do tell them I say that they must be very careful not to take it without the prescription of a physician.

Dr. Wilson—Whenever a physician does tell the patient it always interferes with the treatment of the case. I do not think it a wise thing to do.

Dr. C. M. Selfridge—I had a case this week, and if I told the patient the remedy, her own physician would have given it to her in the mother *tincture* and have spoiled the case for me. The remedy was *Colocynth*.

Dr. J. M. Selfridge then read from *The Organon*, Sections 104–109.

DISCUSSION.

Dr. Martin—This reminds me of an editorial in one of our journals which stated that in the time of Hahnemann there was not known anything like the number of reflex diseases that are now known. We can go a little further and say that Hahnemann did not know about these things. He says here, that while diseases may, in the future, have different names, they will still be the same. In the diseases Hahnemann mentions as being caused by psora, he mentions nearly all the reflex diseases.

Dr. J. M. Selfridge—An editorial of that kind is usually written in the interest of orificial surgery. They want to claim that the little pockets and papillæ cause all the trouble, and that every reflex condition comes from these causes.

Sections 110–118 were read.

DISCUSSION.

Dr. J. M. Selfridge—Speaking of these idiosyncrasies of susceptibility to certain odors. When I was a student I was working at some Dover's Powders (which contain Opium, Ipecac, and Sulphate of Potash), and gave it a blow off my spatula. As I did so my preceptor, Dr. Patterson, walked into the room and was immediately attacked with a bad case of asthma.

Dr. Martin—While triturating some Ipecac, an orderly in the hospital came into the dispensary, and the moment he breathed in the Ipecac, before he could get out of the door, he had an awful attack of asthma. I gave him *Ipecac*$^{6X}$, and in twenty minutes he was all right.

Dr. J. M. Selfridge—Dr. Patterson was not in the habit of having asthma, and this was a pure proving of Ipecac upon him from a few atoms of it around the room.

Section 119 was read.

### Discussion.

Dr. Swayze—Has there been any advancement in allopathic prescribing during the last ten years?

Dr. Martin—The allopaths have made great advancement in the last ten years. They prescribe the single remedy a great deal more now than ever before. Wyeth puts up tablets containing one drop of the single remedy, or a hundreth of a gr. of a remedy.

Dr. Swayze—This is also done in the dosometric system. An allopath gave me Gelsemium according to the dosometric system, and it seemed to burn a hole in my throat, the sensation was so queer.

Dr. Augur—The treatment has *not* improved during the past twenty years. The allopaths are trying to do away with the germs more than anything else. This is the only advancement they have made. Their treatment in diphtheria, pneumonia, or membraneous croup is no better now than when I graduated.

Dr. J. M. Selfridge—They think they have made advancement, for they think they have discovered it in the "horse juice."

Dr. Augur—The homœopaths who prescribe allopathically, prescribe very poorly.

Dr. J. M. Selfridge—They are hurting the cause to do it. The allopaths say they are not practicing what they preach.

Sections 120, 121 were read.

### Discussion.

Dr. Swayze—This is a question to discuss. I cannot accept this off-hand that the drug should be tried upon susceptible and sensitive persons. How would you know that a person was going to be susceptible to an untried drug?

Dr. J. M. Selfridge—You would pick out persons of nervous temperament.

Dr. Swayze—Suppose the drug would act better upon phlegmatic persons.

Dr. Martin—This is only a general rule to follow.

Sections 122–128 were read.

DISCUSSION.

Dr. J. M. Selfridge—Many have taken issue with Hahnemann on the last point mentioned. Hughes, Hale, and Dake are among the persons who, in going over the materia medica they brought out, would discard all remedies proved in this way.

Dr. Ledyard—The Austrian Provers' Union got no effect from Natrum-mur. in the condensed form until the 15th and 30th decimal potencies were used, and the higher they went the better results they had.

Section 129 was read.

Dr. J. M. Selfridge—This answers Dr. Swayze's question—that you cannot tell about the susceptibility of the patient.

The meeting was then declared adjourned, to meet again the first Friday in May, at the office of Dr. George H. Martin, in San Francisco, when the reading of *The Organon* would be commenced at Section 130.

W. E. LEDYARD, *Secretary*.

Reported by Eleanor F. Martin, M. D.

